# Effect of Long-term Incretin-Based Therapies on Ischemic Heart Diseases in Patients with Type 2 Diabetes Mellitus: A Network Meta-analysis

**DOI:** 10.1038/s41598-017-16101-1

**Published:** 2017-11-17

**Authors:** Che-Yi Chou, Ying-Tzu Chang, Jia-Lian Yang, Jiun-Yi Wang, Tsui-Er Lee, Ruey-Yun Wang, Chin-Chuan Hung

**Affiliations:** 1 Kidney Institute and Division of Nephrology, Department of Internal Medicine, China Medical University Hospital, Taichung, 40447 Taiwan; 2 Department of Pharmacy, College of Pharmacy, China Medical University, Taichung, Taiwan R.O.C.; 3 Department of Healthcare Administration, Asia University, Taichung, Taiwan R.O.C.; 4 Office of Physical Education, Asia University, Taichung, Taiwan R.O.C.; 5 Department of Public Health, China Medical University, Taichung, Taiwan R.O.C.; 6 Department of Pharmacy, China Medical University Hospital, Taichung, Taiwan R.O.C.

## Abstract

Patients with type 2 diabetes mellitus (T2DM) experience many cardiovascular complications. Several studies have demonstrated the cardioprotective effects of incretin-based therapies; however, there are few studies on the effects of long-term incretin-based therapies on cardiovascular events. Therefore, the present study conducted a systematic review and network meta-analysis to evaluate the effects of long-term incretin-based therapies on ischaemic diseases. We searched PubMed, CENTRAL, and Clinicaltrial.gov to retrieve randomised control trials reported until December 2016 and enrolled only RCTs with more than a 1-year follow-up. The network meta-analysis was performed using R Software with a GeMTC package. A total of 40 trials were included. Dipeptidyl peptidase 4 inhibitors and glucagon-like peptide-1 agonists were associated with a lower risk of myocardial infarction (MI) than were sulfonylureas (odds ratio [95% credible interval] 0.41 [0.24–0.71] and 0.48 [0.27–0.91], respectively). These results suggested that patients with T2DM receiving long-term incretin-based therapies have a lower risk of MI than do those receiving sulfonylurea-based therapy. These findings highlight the risks of cardiovascular events in patients who receive long-term incretin-based therapies, and may provide evidence for the selection of antidiabetic therapy in the future.

## Introduction

Type 2 diabetes mellitus (T2DM) is a chronic metabolic disorder associated with deficiency in insulin secretion and action. It is a major and growing health problem worldwide and accompanied with many complications that negatively influence the quality of life. One of the most concerned complications is cardiovascular diseases. T2DM patients are associated with two to four fold higher risk of cardiovascular diseases as compared with people under normal glycemic level^1^. The United Kingdom Prospective Diabetes Study (UKPDS)^2^ demonstrated that intensive glycemic control in patients with T2DM may reduce the risk of microvascular outcomes; however, other trials showed that lowering blood glucose intensively did not significantly prevent patients from cardiovascular events^3,4^.

Antidiabetic agents have also been associated with incidences of cardiovascular diseases. Previous studies showed the cardiovascular risk was increased in thiazolidinedione treatments^5,6^. This finding raised the attention of the cardiovascular safety of antidiabetic drugs. In 2008, the US Food and Drug Administration (FDA) revised the approval process of antidiabetic agents and the evaluation of cardiovascular events during phase II and phase III studies were required^7^. Since then, a number of trials have been conducted to clarify the effects of new classes of antidiabetic therapies on cardiovascular events.

Incretin-based therapies are novel medications for T2DM management. There are two types of incretin-based drugs, glucagon-like peptide-1 (GLP-1) receptor agonists and dipeptidyl peptidase-4 (DPP-4) inhibitors. GLP-1 is an endogenous incretin hormone; activation of GLP-1 receptors stimulates insulin secretion and inhibits glucagon. DPP-4 inhibitors control hyperglycemia by blocking DPP-4 enzyme, which degrade incretin hormones-glucose-dependent insulinotropic polypeptide (GIP) and glucagon-like peptide-1^8^.

The potential cardioprotective effects of incretin-based therapies were shown in several studies^9–12^. Although numerous meta-analyses have been conducted to assess the cardiovascular safety of GLP-1 agonists and DPP-4 inhibitors, inconsistent results were report from different reviews, and the long-term outcomes were limited^13–16^. A recent meta-analysis suggest that use of exenatide and saxagliptin may increase the risk of arrhythmia and heart failure, respectively^13^. However, other studies did not demonstrate any differences on cardiovascular risk in comparison with other antidiabetic agents or placebo^14–16^. In addition, the influence of GLP-1 agonists and DPP-4 inhibitors on individual cardiovascular risk remained unclear. Furthermore, the comparisons of GLP-1 agonists versus DPP-4 inhibitors or other antidiabetic agents on cardiovascular outcomes were limited due to the lack of available long-term trial data. Therefore, in the present study we conducted a systematic review and network meta-analysis to comprehensively assess effects of the long-term use of GLP-1 agonists or DPP-4 inhibitors on ischemic heart diseases. The results of the present meta-analysis of randomized control trials may provide an evidence for a decision making of antidiabetic therapy in the future.

## Methods

This systematic review and meta-analysis was conducted according to the guidance of *Cochrane Handbook* ^17^ and following the Preferred Reporting Items for Systematic reviews and Meta-Analyses Extension for Network Meta-analysis (PRISMA-NMA)^18^. The protocol for this systematic review was registered in PROSPERO in November 2016, the registration number is: CRD42016051259.

### Search strategy and selection criteria

We searched for relevant randomize control trials (RCTs) from inception to December 2016 in PubMed and Cochrane central register of controlled trials (CENTRAL) database using medical subject heading (MeSH) terms with “ Glucagon-Like Peptide-1 Receptor/agonists”, or “Dipeptidyl-Peptidase IV Inhibitors”, (see Supplementary Tables S1 and S2). The language was limited to English. We also searched online clinical trials database (ClinicalTrials.gov) to identify additional eligible unpublished data.

Studies met the following inclusion criteria were included in this network meta-analysis: (1) randomized control trials (RCTs), (2) intervention compared DPP-4 inhibitors or GLP-1 agonist against placebo or other antidiabetic agents, (3) adults participants with type 2 diabetes, (4) at least 52 weeks follow-up, (5) Reported the events of coronary artery disease, myocardial infarction or angina in the original articles or on the ClinicalTrials.gov. The definition of these ischemic heart diseases were based on the standard medical terminology, Medical Dictionary for Regulatory Activities (MedDRA). The studies met the following criteria were excluded: (1) duplicate reports; (2) studies have not yet been terminated; (3) observational studies; (4) background treatment was the same as the one arm of studies.

The reference management software EndNote X7 was used to remove the duplicate studies by the “find duplication” function. Full texts were obtained for further review. The potentially relevant studies were identified according to pre-specified inclusion and exclusion criteria by two reviewers independently. Any discordant evaluations resolved by discussion and final consensus.

### Outcome Measures and Data Extraction

Data were extracted independently by two reviewers using the standardized form including study characteristics (author name, publication year, location, sample size, mean age and percentage of male), study design (randomization, blinding, phase and interventions), and outcomes (number of participants with cardiovascular events in intervention group and control group). The primary outcome was any cardiovascular events in T2DM patients who treat with GLP-1 agonists or DPP-4 inhibitors more than one year. In addition, less trials would lead to higher heterogeneity, therefore, we analyzed the events reported in more than three trials. Discrepancies were resolved by discussion until consensus was reached. For any of the unclear information, the corresponding author of that study would be contacted for clarification.

### Risk of bias assessment

The methodological quality assessment was performed by using the Cochrane Collaboration’s tool to assess risk of bias in each trials^19^. The evaluation items including in the present study are random sequence generation, allocation concealment, blinding, incomplete outcome data, selective outcome reporting and the potential bias. Each item was presented as “low risk”, “high risk”, or “unclear risk”. The graphs were synthesis by Review Manager version 5.3 (RevMan 5.3)^20^.

### Statistical Analysis

The evaluation of cardiovascular outcomes was based on the synthesis of data extracted from included trials, then combine direct and indirect comparisons to estimate the overall effects of GLP-1 agonist and DPP-4 inhibitors. In this network meta-analysis we used the random-effects model and conducted in Bayesian framework. The effects of GLP-1 agonists and DPP-4 inhibitors on cardiovascular outcomes were analyzed using the odds ratios (OR) and 95% confidence intervals (CIs). The OR > 1.0 were indicated as higher risk, The CIs which did not include 1.0 was considered to be statistically significant. All the analyses were generated by R Software with GeMTC package^21,22^.

The consistency of network meta-analysis was assessed using the node-splitting models to detect whether the results of direct and indirect comparison were in agreement within treatment loops^23^. The node-splitting models cannot be performed when the outcome which lacked direct or indirect comparison. Thus, we used the analysis of heterogeneity to quantify the degree of heterogeneity by *I* ^2^ calculation. The values of *I* ^2^ > 50% was considered heterogeneity across the trials. To verify the robustness of the results, sensitivity analysis was performed to explore whether any factors might affect overall effect by excluding the heterogeneous studies one at a time then recalculated the overall effect.

## Results

### Study selection and characteristics

**Figure 1:**
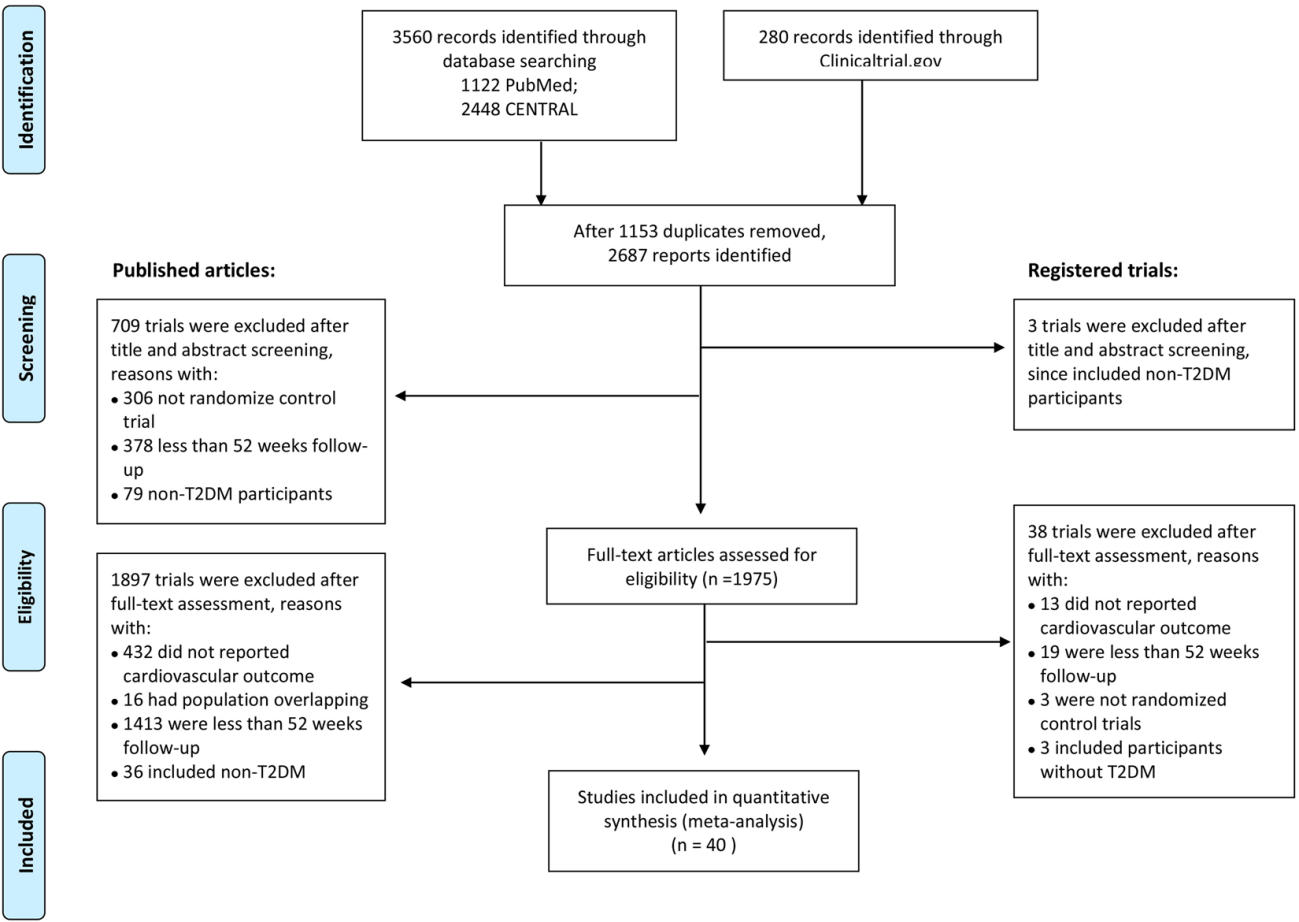
PRISMA-NMA diagram of the literature search. RCTs were identified from PubMed, CENTRAL and Clinicaltrial.gov databases and the searches were done in December 2016. The medical subject heading (MeSH) terms were used in the searching of PubMed and CENTRAL. In the searching of ClinicalTrials.gov, we limited the search for completed RCTs with results. There were 3840 references identified from the databases and a total 40 studies (35 full text publication and 5 unpublished studies) were included in the present study.

A total of 3840 references were identified using the search strategies. After removing 1153 duplicates, 2687 studies were selected through titles and abstracts screening. Full texts were obtained for further evaluation. After pre-screening, 1935 studies were excluded due to unsatisfying the inclusion criteria. Finally, a total of 40 studies which contained 35 full text publication and 5 unpublished studies, fulfilled the inclusion criteria and were reviewed in the present network meta-analysis. The range of publication year was 2007–2016. The flow diagram for results of the electronic search was described in Fig. 1 and the PRISMA NMA Checklist of Items to Include When Reporting A Systematic Review Involving a Network Meta-analysis were represented in Supplementary Table S3.

The characteristics of included studies were shown in Table 1. The mean age of the included 70162 participants was 58.5 years old and the proportion of male was 55.4%. Most included trials were multicenter double-blind randomized design, whereas ten trials were open-label randomized design. There were 26 trials with active-controlled, 13 trials with placebo comparator and 1 trials both with active and placebo comparator. The trials duration ranged from 1 to 3 years.

**Table 1:** Characteristics of included studies.

Study	ClinicalTrials.gov Identifier	Location	Blind	Phase	Follow up (years)	Mean age (years)	Male (%)	Total subject	Treatment	Control
**AWARD-2** ^45^	NCT01075282	Multicenter	Open label	phase III	1.5	56.66	51.3	807	Dulaglutide 0.75–1.5 mg QW for 78 weeks (n = 545)	Insulin SC once daily for 78 weeks; dose titration based on blood glucose measures (n = 262)
**AWARD-4** ^46^	NCT01191268	Multicenter	Open label	phase III	1	59.36	53.5	884	Dulaglutide 0.75–1.5 mg QW for 52 weeks (n = 588)	Insulin SC once daily for 52 weeks; dose titration based on blood glucose measures (n = 296)
**LEAD-3** ^47^	NCT00294723	Multicenter	Double blind	phase III	2	53	49.7	745	Liraglutide 1.8 mg QD for 104 weeks (n = 497)	Glimepiride 8 mg QD for 104 weeks (n = 248)
**Nauck, 2007** ^48^	NCT00082407	Multicenter	Open-label	phase III	1	58.7	48.7	501	Exenatide 5 mcg SQ BID for 4 weeks and followed by 10 mcg for 48 weeks (n = 253)	Insulin SC twice daily; titration to target blood glucose level (n = 248)
**LEAD-2** ^49^	NCT00318461	Multicenter	Double blind	phase III	2	56.7	58.2	1087	Liraglutide 0.6–1.8 mg/day for 104 weeks (n = 724)	Glimepiride 4 mg/day for 104 weeks (n = 242)/ Metformin 1.5–2.0 g/day for 140 weeks (n = 121)
**HARMONY-1** ^50^	NCT00849056	Multicenter	Double blind	phase III	1	55	59.8	301	Albiglutide 30 mg QW (n = 150)	Placebo (n = 151)
**Seino, 2010** ^51^	NCT00393718	Japan	Double blind	phase III	1	58.3	67.3	400	Liraglutide 0.9 mg/day for 52 weeks (n = 268)	Glibenclamide 2.5 mg/day for 52 weeks (n = 132)
**AWARD-1 2014** ^52^	NCT01064687	Multicenter	Double blind	phase III	1	56.5	58.4	979	Dulaglutide 0.75–1.5 mg QW or Exenatide 5 mcg BID for 4 weeks, followed by 10 mcg BID for 48 weeks (n = 837)	Placebo (n = 142)
**HARMONY-4 2014** ^53^	NCT00838916	Multicenter	Open-label	phase III	1	55.5	56.1	745	Albiglutide 30 mg QW, n = 504	Insulin, n = 241
**Seck 2010** ^54^	NCT00094770	NR	Double blind	phase III	2	57.3	60.1	1172	Sitagliptin 100 mg QD (n = 588)	Glipizide 5 mg QD (n = 584)
**Rosenstock 2013** ^55^	NCT00121641	Multicenter	Double blind	phase III	2	53.5	50.1	401	Saxagliptin 2.5–10 mg QD (n = 301)	Placebo (n = 95)
**DeFronzo 2009** ^56^	NCT00121667	Multicenter	Double blind	phase III	4	54.6	50.7	743	Saxagliptin 2.5–10 mg QD (n = 564)	Placebo (n = 179)
**Dobs 2013** ^57^	NCT00350779	Multicenter	Double blind	phase III	1	54.6	58	262	Sitagliptin 100 mg QD (n = 170)	Placebo (n = 92)
**EUREXA 2012** ^58^	NCT00359762	Multicenter	Open-label	phase III	1	56.4	53.6	1019	Exenatide 10 mcg BID, n = 511	Glimepiride 1 mg QD, n = 508
**Bosi 2011** ^59^	NCT00432276	Multicenter	Double blind	phase III	1	55.1	51.5	803	Alogliptin 25 mg QD (n = 404)	Placebo (n = 399)
**Corry 2013** ^60^	NCT00509236	Multicenter	Double blind	phase III	1	59.5	59.7	129	Sitagliptin 25 mg QD (n = 64)	Glipizide 2.5–20 mg QD (n = 65)
**Arjona 2013** ^61^	NCT00509262	Multicenter	Double blind	phase III	1	64.6	57.1	422	Sitagliptin 25–50 mg QD (n = 210)	Glipizide 2.5–20 mg QD (n = 212)
**Gallwitz 2012** ^62^	NCT00622284	Multicenter	Double blind	phase III	2	59.8	61	1551	Linagliptin 5 mg QD (n = 776)	Glimepiride 1 mg QD (n = 775)
**Göke 2013** ^63^	NCT00575588	Multicenter	Double blind	phase III	2	57.6	51.7	858	Saxagliptin 5 mg QD (n = 428)	Glipizide 5–20 mg QD (n = 430)
**Wilson 2013** ^64^	NCT00707993	Multicenter	Double blind	phase III	1	69.9	44.9	441	Alogliptin 25 mg QD (n = 222)	Glipizide 5–10 mg QD (n = 219)
**AWARD-5 2015** ^65^	NCT00734474	Multicenter	Double blind	phase III	2	54	46.7	921	Sitagliptin 100 mg QD (n = 315)	Dulaglutide 0.75–1.5 mg QW (n = 606)
**Barnett 2012** ^66^	NCT00740051	Multicenter	Double blind	phase III	1	56.6	39.9	227	Linagliptin 5 mg QD (n = 151)	Placebo (n = 76)
**Barnett 2013** ^67^	NCT00757588	Multicenter	Double blind	phase IIIb	1	57.3	57.8	455	Saxagliptin 5 mg QD (n = 304)	Placebo (n = 151)
**TECOS 2015** ^68^	NCT00790205	Multicenter	Double blind	phase III	3	65.5	70.3	14523	Sitagliptin 100 mg QD (n = 7332)	Placebo (n = 7339)
**HARMONY-3 2014** ^69^	NCT00838903	Multicenter	Double blind	phase III	2	54.5	47.6	1012	Sitagliptin 100 mg QD (n = 302)	Glimepiride 2 mg QD (n = 307) /Albiglutide 30 mg QW (n = 302) /Placebo (n = 101)
**Del 2014** ^70^	NCT00856284	Multicenter	Double blind	phase III	2	55.4	49.7	2639	Alogliptin 12.5 mg QD (n = 1765)	Glipizide 5–20 mg QD (n = 874)
**Ferrannini 2013** ^71^	NCT00881530	Multicenter	Open- Label	phase II	1.5	58.9	44.3	388	Sitagliptin 100 mg QD (n = 56)	Empagliflozin 10–25 mg QD (n = 332)
**Yki-Järvinen 2013** ^72^	NCT00954447	Multicenter	Double blind	phase III	1	60	52.2	1261	Linagliptin 5 mg (n = 631)	Placebo (n = 630)
**EXAMINE 2013** ^73^	NCT00968708	Multicenter	Double blind	phase III	1.5	61	67.9	5380	Alogliptin 25 mg QD (n = 2701)	Placebo (n = 2679)
**GENERATION 2015** ^74^	NCT01006603	Europe,Mexico	Double blind	phase IIIb/IV	1	72.6	61.8	720	Saxagliptin 5 mg QD (n = 360)	Glimepiride 1 mg QD (n = 360)
**Lavalle-González 2013** ^75^	NCT01106677	Multicenter	Double blind	phase III	1	55.4	46.4	1101	Sitagliptin 100 mg QD (n = 366)	Canagliflozin 100–300 mg QD (n = 735)
**SAVOR-TIMI 53 2013** ^76^	NCT01107886	Multicenter	Double blind	phase IV	2.1	65	67	16492	Saxagliptin 5 mg QD (n = 8280)	Placebo (n = 8212)
**CANTATA-D2 2013** ^72^	NCT01137812	Multicenter	Double blind	phase III	1	56.5	55.9	755	Sitagliptin 100 mg QD (n = 378)	Canagliflozin 300 mg QD (n = 378)
**ELIXA 2016** ^77^	NCT01147250	Multicenter	Open-label	phase III	2.1	60.3	69.4	6068	Lixisenatide 10–20 μg QD, n = 3034	Non-medication, n = 3034
**Roden 2015** ^45^	NCT01289990	Multicenter	Open-Label	phase III	1.5	55	61.3	899	Sitagliptin 100 mg QD (n = 223)	Empagliflozin 10 mg/25 mg QD (n = 448)
	NCT01098539 (139)	Multicenter	Double blind	phase III	1	63.3	53.7	495	Albiglutide 30 mg QW (n = 249)	Sitagliptin 100 mg QD (n = 246)
	NCT01075282(75)	Multicenter	Open-label	phase III	1.5	56.7	51.3	807	Dulaglutide 1.5 mg SC QW for 78 weeks, n = 545	Insulin, n = 262
	NCT01648582(49)	Multicenter	Open-label	phase III	1	54.5	54.5	783	Dulaglutide 0.75–1.5 mg QW for 52 weeks, n = 526	Insulin, n = 257
	NCT01087502(36)	Multicenter	Double blind	phase III	1	66.6	63.4	235	Linagliptin 5 mg (n = 118)	Placebo for 12 weeks and then switch to Glimepiride for further 40 weeks (n = 123)
	NCT01682759 (108)	Multicenter	Double blind	phase III	1	57.7	55.1	751	Omarigliptin 25 mg QW (n = 375)	Glimepiride 1–6 mg QD (n = 376)

QD = once daily. BID = twice daily. QW = once per week. SC = subcutaneous. n = nu mber of participants. Registration number were identify in ClinicalTrials.gov.

**Figure 2:**
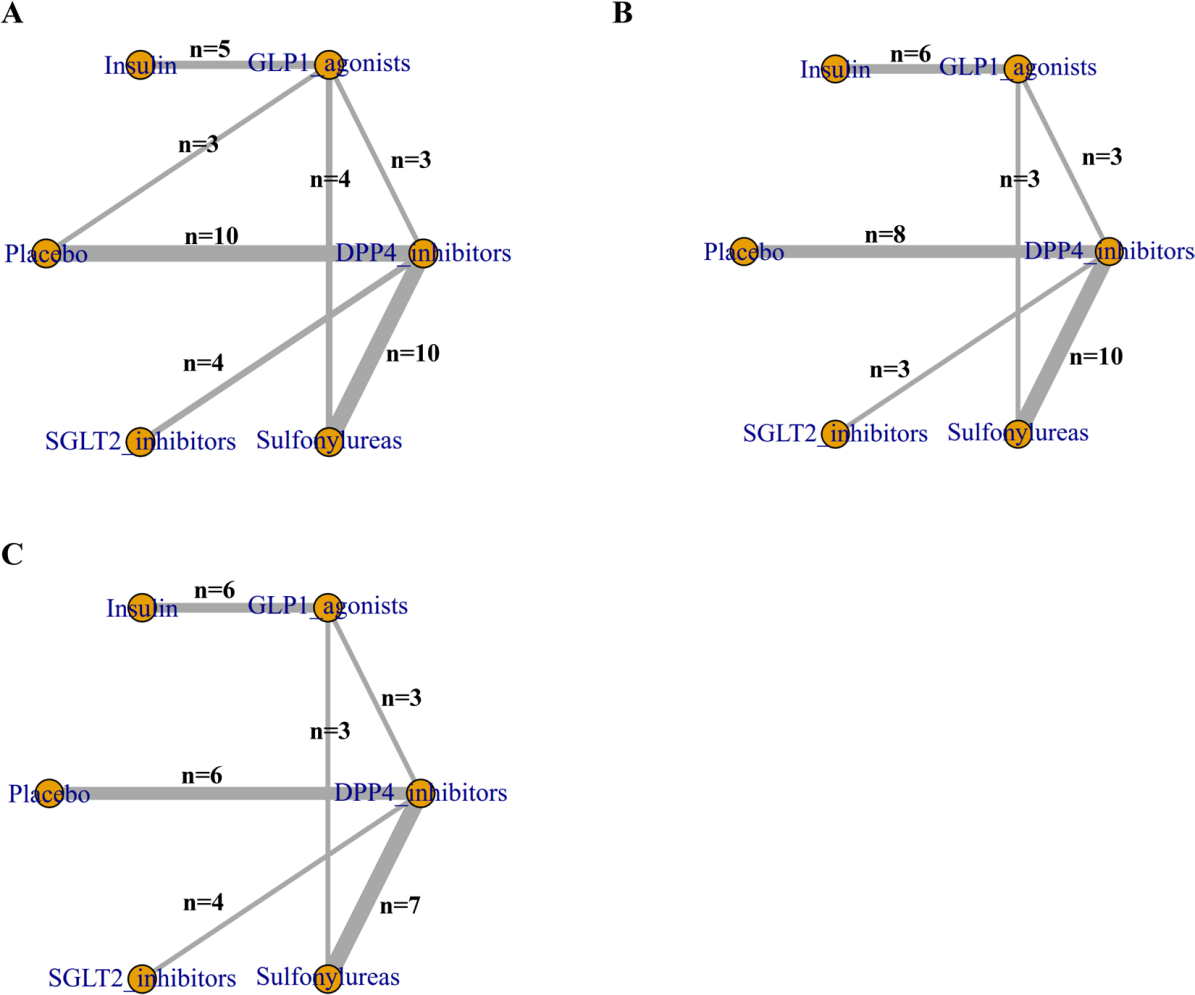
Network of eligible comparisons for (**A**) MI, (**B**) angina and (**C**) CAD. The size circle reflects the number of participants (sample size), and the width of the lines reflects the number of direct comparisons. n = number of trials for the direct comparisons.

Figure 2 showed the network plots of eligible comparisons for myocardial infarction (MI), angina and coronary arterial disease (CAD). There were five classes of antidiabetic agents (DPP-4 inhibitors, GLP-1 agonists, sodium glucose transporter 2 inhibitors, sulfonylureas and insulin) have adequate trials for network- meta-analysis. Both of DPP-4 inhibitors and GLP-1 agonists were indirectly and directly compared with other antidiabetic agents.

### Quality of included studies

**Figure 3:**
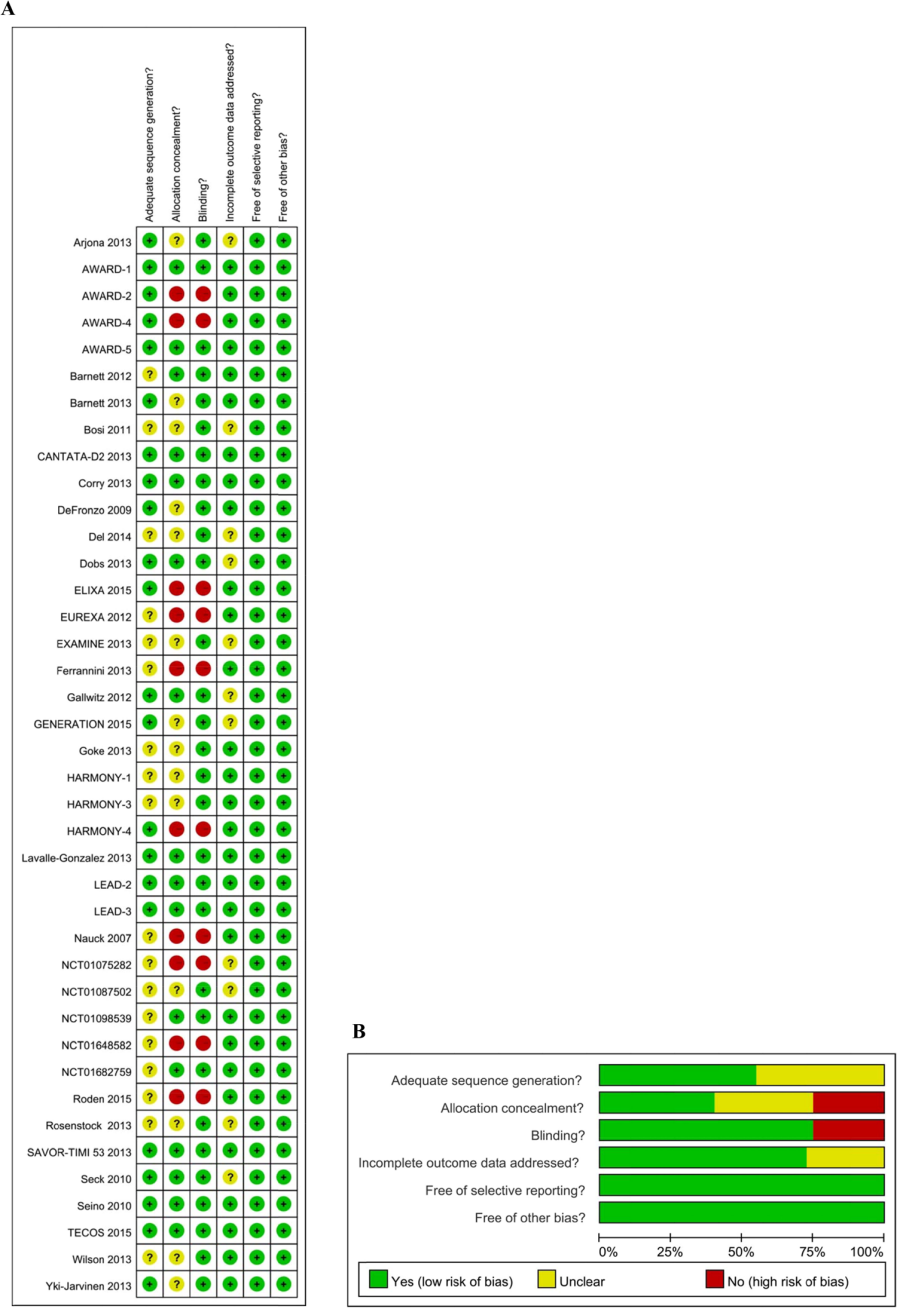
Risk of bias assessment of included trials. The methodological quality assessment was performed by using the Cochrane Collaboration’s tool to assess risk of bias in for included trials. Allocation sequence generation was adequate in most of published trials except open-label trials and the unpublished trials which were judged as unclear. Open-label trials were judged as high risk in the allocation concealment and blinding. Eleven trials were judged as unclear risk of incomplete data, because these studies did not address the outcome analysis. Selective report biases were not identified in the included studies.

Risk of bias assessment of included trials was shown in Fig. 3. Allocation sequence generation was adequate in most of published trials except open-label trials. The unpublished trials were judged as unclear due to the insufficient information of the sequence generation process. The open random allocation schedules were used in open-label trials and were judged as high risk in the allocation concealment and blinding. The outcome measures in these open-label trials were objective parameters, such as blood glucose and cardiovascular events. These outcomes are not self-reported and blinded or not would not influence the results. Therefore, open-label RCTs were included in the present study. Eleven trials were judged as unclear risk of incomplete data, because these trials did not address the outcome analysis. Selective report biases were not identified in the included studies.

### Effects of GLP-1 agonist and DPP-4 inhibitors on myocardial infarction events

**Figure 4:**
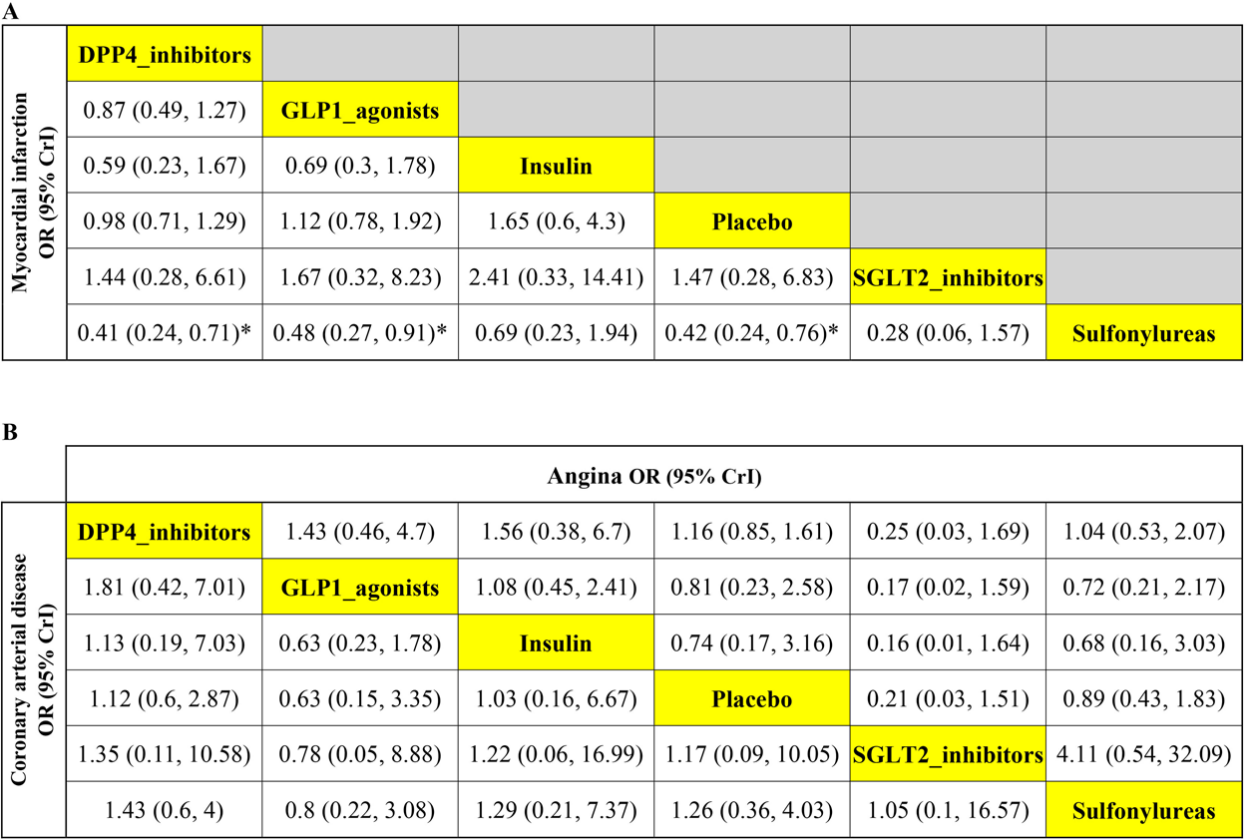
Results of the network meta-analysis for antidiabetic agents in terms of (**A**) MI, (**B**) angina (upper right triangle) and CAD (lower left triangle). Results were presented as OR with 95% CrI, the estimations should read as column-defining treatment compared with the row-defining treatment. The OR below 1 was identified that the column-defining treatment had better effect on the cardiovascular risk. Use of DPP-4 inhibitors and GLP-1 agonists shown high probability with lower risk of myocardial infarction events as compared to use of sulfonylureas. OR = odds ratios. CrI = credible interval. * = 95% CrI did not include 1.

The results of the network meta-analyses for the myocardial infarction events were presented in Fig. 4A. There was no difference effect between use of DPP-4 inhibitors and GLP-1 agonists on the risk of myocardial infarction. On the other hand, pooling data showed use of DPP-4 inhibitors favored lower risk of myocardial infarction events as compared to use of sulfonylureas (OR: 0.41, 95% CrI: 0.24–0.71), and the result of node-splitting analysis did not found any inconsistency between the direct and indirect comparisons (Table 2; p-value = 0.53125).

**Table 2:** Node-splitting analysis of inconsistency within network meta-analysis.

comparison	p-value	OR (95%CrI)
**Myocardial infarction**		
**DPP-4 inhibitors versus GLP-1 agonists**		
direct	0.71125	0.37 (−0.79, 1.6)
indirect		0.099 (−0.34, 0.79)
network		0.12 (−0.27, 0.75)
**DPP-4 inhibitors versus Placebo**		
direct	0.41375	0.0016 (−0.35, 0.33)
indirect		0.39 (−0.54, 1.4)
network		0.014 (−0.26, 0.38)
**DPP-4 inhibitors versus Sulfonylureas**		
direct	0.53125	0.98 (0.38, 1.7)
indirect		0.59 (−0.42, 1.7)
network		0.86 (0.30, 1.4)
**GLP-1 agonists versus Placebo**		
direct	0.38375	−0.037 (−0.61, 0.52)
indirect		−0.43 (−1.4, 0.41)
network		−0.10 (−0.66, 0.26)
**GLP-1 agonists versus Sulfonylureas**		
direct	0.5225	0.49 (−0.45, 1.4)
indirect		0.87 (0.024, 1.7)
network		0.73 (0.072, 1.3)
**Angina**		
**DPP-4 inhibitors versus GLP-1 agonists**		
direct	0.43125	−0.17 (−2., 1.7)
indirect		0.77 (−0.80, 3.1)
network		0.37 (−0.83, 1.6)
**DPP-4 inhibitors versus Sulfonylureas**		
direct	0.495	0.056 (−0.59, 0.80)
indirect		−0.83 (−3.5, 1.6)
network		0.050 (−0.65, 0.67)
**GLP-1 agonists versus Sulfonylureas**		
direct	0.50125	−0.68 (−2.6, 0.75)
indirect		0.18 (−1.7, 2.3)
network		−0.29 (−1.7, 0.83)
**Coronary arterial diseases**		
**DPP-4 inhibitors versus GLP-1 agonists**		
direct	0.02875	0.78 (−0.85, 2.8)
indirect		−2.2 (−4.8, −0.28)
network		−0.65 (−1.9, 0.60)
**DPP-4 inhibitors versus Sulfonylureas**		
direct	0.02	−0.70 (−2., 0.24)
indirect		2.5 (−0.036, 5.7)
network		−0.34 (−1.3, 0.56)
**GLP-1 agonists versus Sulfonylureas**		
direct	0.015	1.6 (−0.11, 3.9)
indirect		−1.5 (−3.9, 0.36)
network		0.28 (−1.0, 1.5)

p < 0.05: significant inconsistency between direct and indirect evidence.

In terms of GLP-1 agonists, a trend of lower risks effects on myocardial infarction risk as compared with sulfonylureas were also observed (Fig. 4A; OR: 0.48, 95% CrI: 0.27–0.91). There were no inconsistency between the direct and indirect comparisons from of node-splitting analysis (Table 2; p-value = 0.5225). In the heterogeneity analysis, global *I*-squared did not identified any heterogeneity across the studies (Table 3; global *I* ^2^ = 15.67).

**Table 3:** Analysis of heterogeneity.

t1	t2	i2.pair	i2.cons	incons.p
**Myocardial infarction**				
**Per-comparison I-squared:**				
DPP4_inhibitors	GLP1_agonists	0	0	0.68
DPP4_inhibitors	Placebo	32.12	24.82	0.91
DPP4_inhibitors	SGLT2_inhibitors	44.39	44.38	NA
DPP4_inhibitors	Sulfonylureas	15.62	5.24	0.58
GLP1_agonists	Insulin	44.14	44.27	NA
GLP1_agonists	Placebo	0	0	0.86
GLP1_agonists	Sulfonylureas	0	0	0.56
**Global I-squared:**				
		19.40	15.67	
**Angina**				
**Per-comparison I-squared:**				
DPP4_inhibitors	GLP1_agonists	67.00	52.20	0.47
DPP4_inhibitors	Placebo	0	0	NA
DPP4_inhibitors	SGLT2_inhibitors	46.17	47.17	NA
DPP4_inhibitors	Sulfonylureas	6.98	0	0.76
GLP1_agonists	Insulin	33.38	33.05	NA
GLP1_agonists	Sulfonylureas	61.08	33.03	0.53
**Global I-squared:**				
		30.56	26.56	
**Coronary arterial diseases**				
**Per-comparison I-squared:**				
DPP4_inhibitors	GLP1_agonists	0	19.84	0.05
DPP4_inhibitors	Placebo	0	0	NA
DPP4_inhibitors	SGLT2_inhibitors	34.72	34.67	NA
DPP4_inhibitors	Sulfonylureas	32.50	28.73	0.16
GLP1_agonists	Insulin	31.44	30.88	NA
GLP1_agonists	Sulfonylureas	0	13.46	0.09
**Global I-squared:**				
		19.96	16.26	

t1: treatment 1, t2: treatment 2, i2.pair: i-square of pair-wise meta-analysis, i2.cons: i-square of network meta-analysis, incons.p: inconsistency p-values for pair-wise and network meta-analysis NA: not applicable.

### Effects of GLP-1 agonist and DPP-4 inhibitors on angina events

The comparisons among five classes of antidiabetic agents, incretin-based therapies did not show significant effects on the risk of angina as compared with other antidiabetic agents or placebo (Fig. 4B upper right triangle). In addition, there was no different effect between GLP-1 agonists and DPP-4 inhibitors on angina events (OR: 1.43, 95% CrI: 0.46–4.7). The result of node-splitting analysis did not found any inconsistency between the direct and indirect comparisons (Table 2). There was no significant heterogeneity across the studies regarding angina events (Table 3; global-*I²* = 26.56%).

### Effects of GLP-1 agonist and DPP-4 inhibitors on coronary artery disease events

Coronary artery disease risk was reported in twenty-nine RCTs. Patients with incretin-based therapies did not show superior effect on coronary artery disease risk whether compared with other antidiabetic agents or placebo (Fig. 4B lower left triangle). When further compared between GLP-1 agonists and DPP-4 inhibitors, no significant difference was detected (OR: 0.55, 95% CrI: 0.14–2.41). The results of node-splitting analysis showed inconsistency between the direct and indirect comparisons (Table 2), however, the degree of heterogeneity was low across the RCTs regarding coronary artery disease events (Table 3; global-*I*² = 16.26%).

## Discussion

The present network meta-analysis comprehensively analysed 40 RCTs that reported the occurrence of cardiovascular events in patients receiving antidiabetic treatment for more than 1 year. The direct and indirect comparison results indicate that patients with T2DM receiving long-term incretin-based therapies are not at an increased risk of angina or coronary arterial disease. By contrast, DPP-4 inhibitors or GLP-1 agonists are associated with a lower risk of MI than are sulfonylureas.

Several systematic reviews and meta-analyses have evaluated the associations between antidiabetic treatment and cardiovascular events. Some of these studies have suggested that sulfonylurea use results in an increased risk of cardiovascular events or death^24–26^. Evidence has also suggested that DPP-4 inhibitors or GLP-1 agonists exert cardioprotective effects in patients with T2DM^27–34^. The present network meta-analysis demonstrates that patients with T2DM receiving long-term incretin-based therapies are at a low risk of MI. Additionally, in accordance with the present study, prior research has demonstrated the beneficial effects of GLP-1 agonists or DPP-4 inhibitors on MI, and has suggested that GLP-1 agonists improve myocardial blood flow and reduce regional infarction^35^. Furthermore, DPP-4 inhibitors are recognised as reducing the risk of MI compared with a placebo^12,36^.

These findings are supported by animal models and *in vivo* studies. In a mouse model, GLP-1 improved functional recovery after ischaemic injury by increasing cardiomyocyte viability and coronary vasodilatation^10^. The development of atherosclerotic lesions was also suppressed and cardiac infarct size was decreased in mice pretreated with GLP-1^37,38^. In another study, a GLP-1 analogue exerted protective effects against cardiomyocyte hypertrophy, interstitial fibrosis, and myocardial inflammation^39^. These effects were associated with the reduction of inflammation and oxidative stress, which are risk factors for ischaemia. In a clinical study, lower plasma GLP-1 levels were observed in patients with coronary artery disease^40^. Compared with sulfonylurea use, the use of GLP-1 agonists significantly improved several cardiovascular risk factors, including body weight, waist circumference, and blood pressure, in patients with T2DM^41^.

Several studies have also reported controversial results regarding the cardioprotective effects of incretin-based therapies. A recent meta-analysis revealed no differences in the risk of MI between patients with T2DM receiving incretin-based therapies and those receiving a placebo (OR: 0.95, 95% CI: 0.88–1.03, P = 0.18)^16^. In other meta-analysis studies, no significant differences have been observed in the risk of cardiovascular events between incretin-based therapies and other antidiabetic agents^14,15^. However, these studies could not clarify the influence on individual cardiovascular outcomes. Furthermore, some of the included RCTs had a relatively short-term follow-up period, and therefore may have underestimated the actual benefits because the effects of a decreased risk of cardiovascular events may require long-term study (e.g., a 52-week follow-up) to be observed. In addition, there are few studies on the comparison of incretin-based therapies with each class of antidiabetic agents. One previous network meta-analysis compared the efficacy of oral antidiabetic drugs on cardiovascular events and mortality^42^; however, the results did not reveal differences in the effects of DPP-4 inhibitors and other antidiabetic agents or a placebo on MI in patients with T2DM. Elsewhere, patients receiving sodium glucose cotransporter-2 inhibitors were found to have a lower risk of MI than were those receiving a placebo (RR: 0.77, 95% CI: 0.63–0.93) or DPP-4 inhibitors (RR: 0.75, 95% CI: 0.60–0.94)^42^. The present study compared the potential cardioprotective effects of long-term incretin-based therapies and other antidiabetic drugs, namely DPP-4 inhibitors or GLP-1 agonists, or a placebo. We did not include RCTs on any other antidiabetic drugs to avoid inconsistent results.

The present results did not reveal any effects of GLP-1 agonists and DPP-4 inhibitors on the risk of angina and coronary artery disease in patients with T2DM. Coronary artery disease is caused by atherosclerosis, which can be asymptomatic. Fatty plaques accumulate on the coronary artery lumen, resulting in decreased heart blood flow, and symptoms such as chest pain, heartburn, or heart attack indicate the possible occurrence of myocardial ischaemia, angina, and MI. Therefore, this discrepancy in results might be because coronary artery disease and angina are imperceptible symptoms before MI that may have progressed to MI at diagnosis^43^. In addition, inconsistencies in direct and indirect comparison results regarding the risk of coronary artery disease may have been caused by the limited number of RCTs. In short, additional studies must be explored to verify the results.

The present study has several notable strengths. First, we used rigorous criteria to identify and include data from RCTs to minimise methodological bias resulting from the problematic quality of evidence, which has been observed in previous reviews. Second, the most comprehensive RCTs were included in this network meta-analysis. Apart from published data, additional unpublished data were identified from the ClinicalTrials.gov database. Obtaining data from unpublished trials can help researchers avoid publication bias, which is a major concern when attempting to establish the validity of meta-analyses. Third, the subgroup analysis of ethnic characteristics could not be performed because the results of different ethnicity were provided from the database of the sponsor and would not be able to obtain from the published studies^44^. We carefully identified the include data and used the random-effects model to minimize methodological bias. Fourth, we strictly ensured data authenticity by carefully evaluating the consistency of data from journal publications and trial registers, which may substantially reduce the risk of outcome-reporting bias. Nevertheless, the present study has some limitations. First, we only included RCTs published in English, and therefore may have excluded related studies published in non-English languages. However, we have included most of the major published trials as well as those from the ClinicalTrials.gov database to reduce bias. Second, some trials might not have reported all outcomes in their publications. However, we obtained the relevant information from their registration data in the ClinicalTrials.gov database. Finally, only a few trials had more than a 1-year follow-up. This limitation reflects the insufficiency of the currently available RCTs, and therefore warrants additional investigation.

In conclusion, the present systematic review and network meta-analysis comprehensively compared the risks of MI, angina, and coronary arterial disease in patients with T2DM receiving incretin-based therapies and other antidiabetic agents. This study demonstrates that more than 1 year of DPP-4 inhibitor or GLP-1 agonist use is associated with a lower risk of MI than is sulfonylurea use in patients with T2DM. Additional studies with a larger sample size, longer follow-up, and novel antidiabetic agents are recommended to derive definitive conclusions regarding the major clinical benefits and risks of these therapies.

### Data availability

The datasets generated during and/or analyzed during the current study are available from the corresponding author on reasonable request.

## Electronic supplementary material

Supplementary Information
